# Dietary preference of cheetahs (*Acinonyx jubatus*) in south‐eastern Kenya

**DOI:** 10.1002/ece3.8556

**Published:** 2022-03-18

**Authors:** Noreen M. Mutoro, Robert Chira, Nathan Gichuki, Edward Kariuki, Jonas Eberle, Jan Christian Habel, Mary Wykstra

**Affiliations:** ^1^ 27257 Evolutionary Zoology Department of Environment and Biodiversity University of Salzburg Salzburg Austria; ^2^ 107854 School of Biological Sciences University of Nairobi Nairobi Kenya; ^3^ Action for Cheetahs in Kenya Nairobi Kenya; ^4^ 137722 Kenya Wildlife Service Nairobi Kenya

**Keywords:** prey abundance, prey availability, prey preference, rangelands, scat analyses

## Abstract

The conversion of natural ecosystems due to anthropogenic activities has led to the destruction of natural habitats and to the deterioration of habitat quality. Top predators particularly respond sensitively to changes in habitat structures, including the availability of prey. The cheetah *Acinonyx jubatus* prefers small‐medium‐sized, wild ungulate prey due to the cheetah's morphological adaptations. However, the majority of the species’ population is found beyond protected areas, where habitat structures, species abundances, and community composition are highly influenced by human activities. Only few studies have analyzed the diet preference of cheetahs in relation to prey availability and abundance for rangelands beyond protected areas in Eastern Africa. The study aimed to determine cheetah prey preference in the rangelands of south‐eastern Kenya based on scat analyses. We compared dietary preference of cheetah with prey availability. For this purpose, we conducted standardized game counts. We analyzed 27 cheetah scat samples collected across the same study area where we also conducted game counts. We found that Grant's gazelle Gazella granti contributed the highest portion of cheetah's diet, although Thomson's gazelle Gazella thomsonii was the most abundant medium‐sized ungulate prey in the study areas. We also recorded two primate species, yellow baboon Papio cynocephalus and vervet monkey Chlorocebus pygerythrus, as well as the rock hyrax Procavia capensis in the cheetah diet. These species have never been documented as cheetah prey before. Furthermore, our results document livestock as potential prey for cheetahs. These observations underline that cheetah use diverse prey in rangelands outside protected areas, and that the abundance of specific prey does not influence cheetah prey preference.

## Graphical abstract

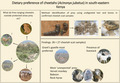

## 1 INTRODUCTION

**FIGURE 1:**
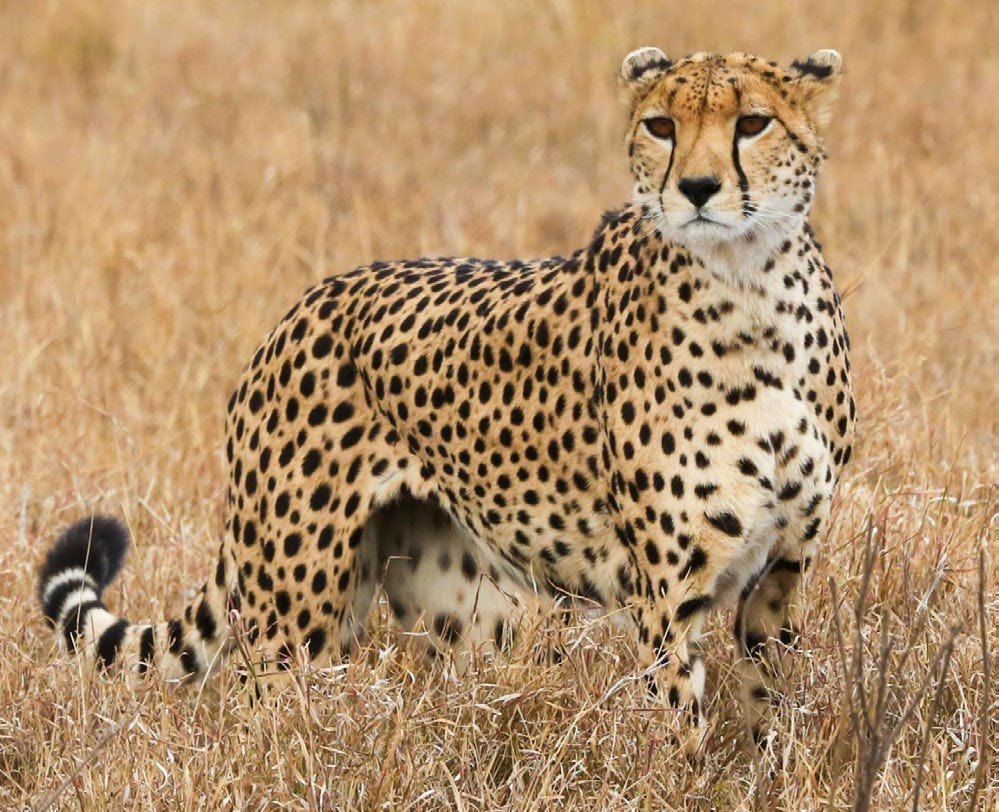
Cheetah, *Acinonyx jubatus*, in East African savannah. Photo credit Denise Wagner

The main driver of biodiversity loss is habitat destruction and the deterioration of habitat quality (Pimm & Raven, 2000). Anthropogenic activities such as agriculture, resource extraction, and urban sprawling can dramatically alter the structure and quality of natural habitats (Doligez & Boulinier, 2008; Laurance, 2010). This transformation of habitats significantly impacts plant and animal species, and may lead to changes in species densities and species composition as well as the extinction of taxa over time (Laurance, 2010). Top predators in particular are affected by these environmental changes, as most of them demand large habitats and specific prey, such as the cheetah (Figure 1) across major parts of Africa (Kuijper et al., 2016).

Carnivore dietary studies are fundamental for a better understanding of predator ecology and the effects of predators on ecosystems (Monterroso et al., 2019). They can also inform conservation and management of both predators and their prey (Shehzad et al., 2012). Diet can be assessed by various methods though each is subjected to different biases (Monterroso et al., 2019). Opportunistic and direct observation of kills is impractical in areas with dense vegetation or when studying elusive species, which cover wide ranges and occur in low densities, especially outside protected areas (Boast et al., 2016; Marker et al., 2003). Quantification of undigested prey, especially through scat analyses is a method widely used to determine food habits of carnivores. This method can provide both qualitative and quantitative diet information of a species (Klare et al., 2011). Potential problems relating to scat analyses is with the accurate identification of carnivore scat in the field and the accurate identification of the prey taxa due to unidentifiable items such as hair and bone fragments (Shehzad et al., 2012). In addition, differential digestibility of food items can lead to a biased conclusion of the dietary estimates (Marker et al., 2003; Monterroso et al., 2019).

Cheetahs exist in a mosaic consisting of protected natural habitats, rural and communal lands with livestock farming, game farms, agricultural croplands, and settlements (Durant et al., 2017; Jeo et al., 2018). This species is a highly efficient hunter that is able to survive in areas with comparatively low prey densities (Farhadinia et al., 2012). Sufficient access to prey is of key relevance to cover its fundamental energetic requirement, which determines species’ fitness (Jeo et al., 2018). Cheetahs are also opportunistic predators and can feed on a wide range of species, but mainly prey on wild ungulates with body masses between 23 and 56 kg (Hayward et al., 2006). However, cheetah prey composition locally varies and strongly depends on the availability and abundance of prey (Farhadinia et al., 2012; Hayward et al., 2006).

Cheetah dietary habits have been documented for populations living in farmlands and rangelands beyond protected areas in Southern Africa and Iran, where cheetahs have been observed to prey on both wild game and domestic animals (Boast et al., 2016; Farhadinia et al., 2012; Marker et al., 2003). However, little is known about cheetah dietary habits in the rangelands outside protected areas in Eastern Africa, especially Kenya, where the majority of the current global population of cheetah is found and where land use and land cover change might have affected cheetah resource utilization and changes in prey composition. More than 80% of the cheetah population in Kenya mainly occurs in community and private lands outside protected areas, where population densities of potential wild ungulate prey species have strongly decreased during the past decades (KWS, 2010; Ogutu et al., 2016).

To evaluate population viability and population trends of cheetahs, a better understanding of the use of its diet resources is crucial (Farhadinia et al., 2012). In this study, we investigated the diet preference of free‐ranging cheetahs in south‐eastern Kenya. We performed scat analyses on 27 cheetah scats collected in eight ranches of the greater Athi Kapiti Plains. This region is characterized by smallholder farming, commercial ranging, and wildlife conservation (Imbahale et al., 2008; Olang & Njoka, 1987). To study cheetah diet preferences, we conducted standardized game counts and concurrently collected scats in the same area. Based on these data we study (i) abundance and composition of potential prey species for cheetahs and (ii) diet preference of cheetah in relation to abundance of prey.

## 2 MATERIAL AND METHODS

### 2.1 Study area

**FIGURE 2:**
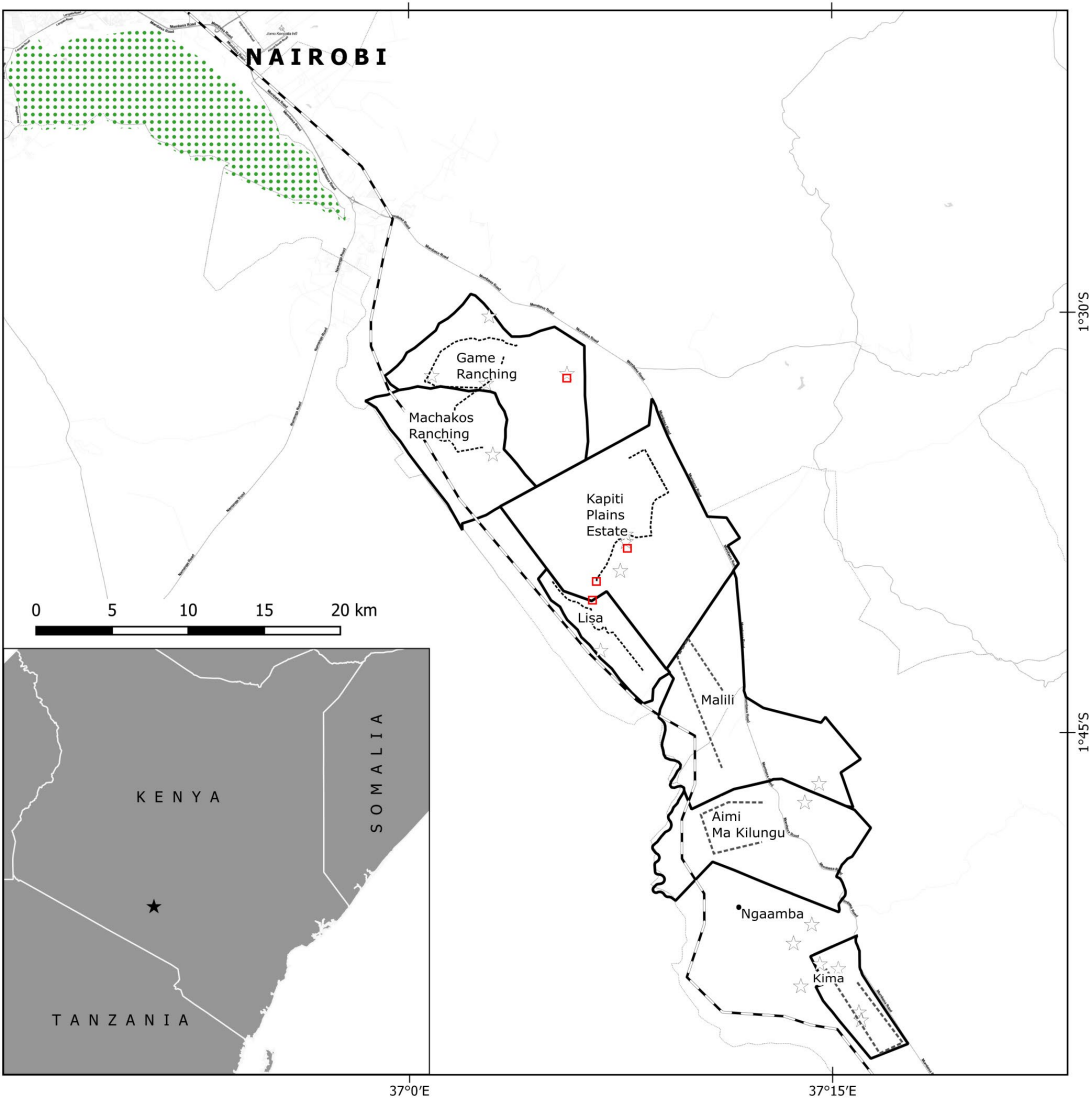
Location of the study are in south‐eastern Kenya (small inlet map), and the eight ranches indicated by names and respective borders. Cheetah scats are visualized by black stars, transects set for driving game counts are displayed as gray lines and scent marking sites as squares

We conducted our study in the following eight ranches: Kima, Malili, Aimi Ma Kilungu, Ngaamba, Game Ranching, Lisa, Machakos Ranching, and Kapiti Plains Estate, all located in the greater Athi Kapiti Plains in south‐eastern Kenya (S 1.30.25, E 37.0.34 and S 1.42.44, E 37.12.0; Figure 2). These ranches cover approximately 450 km^2^. The region is a semi‐arid savannah region with a mean annual rainfall of 510 mm, which is divided into two rainy seasons, with long rains in March–April and short rains in September–October (Jaetzold et al., 2006). The vegetation predominantly consists of Themeda triandra, a tufted perennial grass with a height of 50–150 cm that is valuable for grazers, and “Themeda Acacia” or “Themeda Balanites” wooded grassland (Kinyua et al., 2000). Apart from livestock, this landscape harbors several wildlife species, including cheetah (Kinyua et al., 2000; Wambua, 2008). The estimated cheetah population is 16–20 individuals, depending on the number of cubs. Telemetry studies approved home ranges of approximately 108 km^2^ with core home ranges of about 23 km^2^ (Wykstra, 2007).

This study region has experienced major land use and land cover change between 1980 and 2010, which negatively affected habitat quality (Kiarie, 2014; Wambua, 2008). During the colonial period, commercial ranches were set up in this region. After independence, most colonial settlers offered their ranches for sale to the local people (Olang & Njoka, 1987). Most of these ranches were subsequently sub‐divided into smaller parcels for smallholder farming (Kiarie, 2014; Olang & Njoka, 1987). Changes in land tenure system from cooperative ranching to individual land ownership dramatically shifted land use patterns and management practices to small‐scale agro pastoral land use systems, which have been associated with environmental degradation of rangelands in Kenya (Kiarie, 2014; Ogutu et al., 2016; Olang & Njoka, 1987). Also, decline in wildlife densities have been observed in sub‐divided ranches (Wambua, 2008). Some of the ranches (Game Ranching, Lisa Ranch, Machakos Ranching, and Kapiti Plains Estate) have been not sub‐divided; these are mainly individually owned and the main land use is livestock keeping under ranching and wildlife conservation (Kiarie, 2014; Kinyua et al., 2000).

### 2.2 Assessment of potential prey

To study the species composition and abundance of potential cheetah prey, we surveyed wildlife along eight transect routes (9.3 ± 6.1 km) within the study area between July 2012 and December 2013 (Figure 2). The length and position of the transects were based on the size of the ranches, the type of vegetation and accessibility to ensure a comprehensive survey during both the wet and dry season. The transects only covered a quarter of the ranches but they cut across the representative vegetation types. A uniform width of 400 m was established along each transect and only animals within a maximum range of 200 m from either side of the transect line were recorded (Wambua, 2008). Each transect was surveyed twice per month, with vehicle speed maintained at 15 km/h. We conducted counts during morning (6–10 a.m.) and evening (7:30–9 p.m.) using a strong spotlight, which we swept from side to side up to a 90° angle from the car to spot an animal's eye glare. We still recorded diurnal species in our evening game counts in addition to other nocturnal species such as hares. For each sighting we recorded species name, age, sex, and the number of individuals of a respective species as well as the exact GPS location. We considered both, diurnal, and nocturnal species. All game counts were conducted by the same wildlife expert. The relative abundance of all potential cheetah prey species was then calculated (Craig et al., 2017).

### 2.3 Collection and analysis of scat

Scat was collected between July 2012 and December 2013 from four known cheetah scent‐marking sites where cheetahs had been repeatedly sighted on camera traps. The scent marking sites were located in Game Ranching, Lisa Ranch, and Kapiti Plains Estate (Figure 2) and were visited once per month. Scats were also collected opportunistically along roads, near dams and in areas where cheetahs had been previously sighted in the study area. In order to prevent recollection of scat in scent marking sites, we took photographs of the scat that was left behind after collection. Cheetah scat was first differentiated from that of other sympatric carnivores such as leopard in the field based on different features (color, shape, size, presence of tracks, location, and content). With the closer examination of the scat content, a species assignment was carried out since in cheetah scat there are mainly hair, bones, and sometimes insects, but with other carnivorous mammals like bat‐eared foxes, also insects (e.g., elytra from dung beetle) and seeds of plants can be found. This fact allows the exclusion of other potential carnivores. Species identity was verified in the laboratory based on the presence of cheetah hairs (mainly due to grooming as instances of cannibalism in cheetahs are rare) in the scat (Boast et al., 2016; Lovari et al., 2009; Marker et al., 2018).

In the next step, we analyzed the diet composition based on hairs and bones in the scats. For that, scat samples were individually placed in nylon stockings and washed through two complete regular cycles in a conventional washing machine without the use of any detergents (Marker et al., 2003). This washing process left only hairs, bones, teeth, and hooves in the stockings. The nylon stockings with the remaining undigested material from the scat was subsequently dried in the sun. The dried remains were spread evenly into a dissecting pan with a grid of 10 equal squares and one hair was randomly sampled from each square. Teeth, bones, hooves, insects, and any other identifiable remains were separated from the hair. A scale cast of each of the 10 hairs from a scat sample was obtained by mounting hair on a glass side using clear nail polish for 30 min to obtain the impression of the scale (Chattha et al., 2015). Nomenclature of the scale pattern followed that of Keogh (1983). A whole mount of the same hairs was prepared by placing each hair parallel on a microscope slide. Two drops of gelatin were added and a cover slip was placed on the hairs (Chattha et al., 2015). At least four slides were made for each scat. Features of the cuticle and cortex/medulla were examined under a Leica microscope at 400× magnification. Both predator and prey were identified to species level by comparison with a reference collection of microphotographs of the structure of the cuticle and medulla of hairs from back, belly, shoulder, and hip of potential prey species at a magnification of 400× using sample slides made from all potential prey species in the study area. Hairs used in the reference hair catalog were obtained from museum specimens, carcasses of domestic and wild animals in the study area and sample slides from a carnivore scatology study by Ogara et al. (2010). Frequency of occurrence of a prey species gives an indication of the importance of the prey type in providing a regular food source (Bowland & Perrin, 1993). The frequency of occurrence of each prey species was calculated by dividing the number of scats which contained that species by the total number of scats (Craig et al., 2017).

Prey mass of each species was obtained from three‐quarters of the mean female body mass of that species in order to account for calves and sub‐adults eaten by cheetah (Hayward et al., 2006). We grouped prey species into live weight categories of <23, 23–56 and >56 kg as was used by Hayward et al. (2006). The mean female body mass of different species was obtained from Kingdon (2011).

### 2.4 Statistics

Jacobs Index (Jacobs, 1974) was used to determine the degree of preference for each prey species depending on their abundance:

$$D=\frac{r\;‐\;p}{r\;+\;p\;‐2\text{rp}}$$

where *r* is the proportion of prey species in the cheetah scat and *p* is the proportion of available prey (abundance). The selectivity index D varies from +1 to −1, where +1 indicates maximum preference; −1 indicates maximum avoidance and a null value indicates proportional use of the prey, in relation to its availability.

Following Neu et al. (1974), we performed a chi‐square goodness‐of‐fit test in R (v.3.6.1, R Core Team, 2020) to assess if there was an overall significant preference or avoidance of prey species in relation to abundance. Prey abundance deduced from game counts was scaled to represent expected probabilities. In both analyses, we only included prey species whose abundance was recorded during the game counts, because the nature of the chi‐square goodness‐of‐fit formula,

$$\chi^{2}=\sum \frac{{\left(O‐E\right)}^{2}}{E}$$

where *O* are the observed proportions and *E* are the expected proportions, leads to infinite chi‐squared values if *E* becomes 0 and thus always to high statistical significance.

The selection ratio $\left({\hat{w}}_{i}\right)$ for a prey species *i* was estimated as (*o_i_*/*π_i_*) where *o_i_* is the proportion eaten and *π_i_* is the proportion in the prey population (Höner et al., 2002;Kissui & Parker, 2004; Manly et al., 2002). The standardized selection ratio (*B_i_*) is calculated as ${\hat{w}}_{i}/\left(\Sigma^{i=1}{\hat{w}}_{j}\right)$ and estimates the probability of a particular prey species *i* being selected if all prey types were equally available; standard errors and χ^2^ statistics were determined following Manly et al. (2002).

## 3 RESULTS

During the study period, we made 12,482 observations of potential cheetah prey species. The species observed ranged in size from small (<23 kg), medium (23–56 kg) prey, and large‐sized prey (>56 kg; Tables 1 and 2).

**TABLE 1:** Prey species composition and abundance in the study area recorded during day and night game counts in 2012 and 2013

Species	Day counts	Night counts	Total abundance
Bushbuck, Tragelaphus sylvaticus	0	2	2
Cape hare, Lepus capensis	11	351	362
Dik‐dik, Madoqua kirkii	7	28	35
Duiker, Sylvicapra grimmia	3	42	45
Eland, Tragelaphus oryx	26	148	174
Gerenuk, Litocranius walleri	10	6	16
Giraffe, Giraffa camelopardalis	136	55	191
Grant's gazelle, Gazella granti	326	310	636
Impala, Aepyceros melampus	206	159	364
Hartebeest, Alcephalus busephalus	740	852	1250
Lesser kudu, Tragelaphus imberbis	2	12	14
Reedbuck, Redunca redunca	1	0	1
Spring hare, Pedetes surdaster	2	691	693
Steenbok, Raphicerus campestris	6	31	37
Thomson's gazelle, Gazella thomsoni	667	541	1208
Warthog, Phacochoerus africanus	6	2	8
Wildebeest, Connochaetes taurinus	1981	2841	4822
Zebra, Equus burchellii	1355	1268	2623

**TABLE 2:** Number of cheetah scats (out of 27 scat samples), which contained hairs from each prey item, the frequency of occurrence of prey from cheetah scats, and the relative abundance (%) of prey (out of 12,482 observations) assessed in the study area

Species	Weight category (kg)	*N* scat	Frequency of occurrence (%)	Relative abundance (%)
Grant's gazelle	23–56	7	25.93	5.10
Cape hare	<23	6	22.22	2.90
Goat, Capra hircus	23–56	5	18.52	–
Bushbuck	23–56	5	18.52	0.02
Spring hare	<23	4	14.81	5.55
Sheep, Ovis aries	23–56	3	11.11	–
Zebra	>56	3	11.11	21.01
Giraffe	>56	2	7.41	1.53
Hartebeest	>56	2	7.41	10.01
Wildebeest	>56	2	7.41	38.63
Baboon, Papio cynocephalus	<23	2	7.41	–
Impala	23–56	2	7.41	2.92
Rock hyrax, Procavia capensis	<23	3	11.11	–
Cow, Bos Taurus	>56	1	3.70	–
Common duiker	<23	1	3.70	0.36
Thomson's gazelle, Gazella thomsoni	<23	1	3.70	9.68
Warthog, Phacochoerus africanus	23–56	1	3.70	0.06
Vervet monkey, Chlorocebus pygerythrus	<23	1	3.70	–
Steenbok	<23	1	3.70	0.30
Lesser kudu	>56	1	3.70	0.11
Giant rat, Crycetomis emini	<23	1	3.70	–

A total of 262 carnivore scat samples were collected in the field. Twenty‐seven (10.3%) of them were identified as cheetah scat because they contained cheetah hairs. Cheetah prey composition comprised of 21 different prey species (Table 2). The most frequent prey species consumed by cheetah was Grant's gazelle, followed by Cape hare Lepus capensis and domestic goat Capra hircus and bushbuck Tragelaphus sylvaticus, which had equal frequencies (Table 2).

**FIGURE 3:**
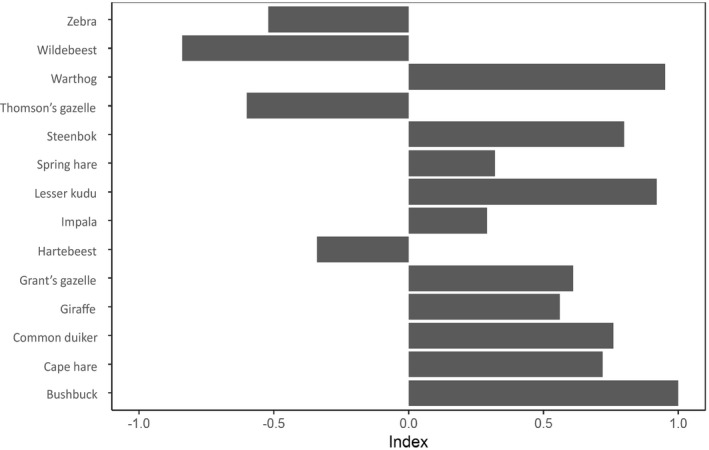
Prey preference of cheetahs in south‐eastern Kenya using the Jacob's Index for preference or avoidance. Values >0 indicate that a prey species was killed more than expected, according its availability (preference), values <0 indicate that a prey species was killed less than expected (avoidance) according to the availability of the respective prey species

Prey availability did impact cheetah’s prey preference in our study area (χ^2^ = 4149.8, df = 13, *p* < .001). Cheetah showed preference for warthog (selectivity index = 0.95), bushbuck (selectivity index = 1.0), and lesser kudu (selectivity index = 0.92). It also showed avoidance of large prey species like wildebeest Connochaetes taurinus (selectivity index = −0.84), common zebra (selectivity index = −0.52), and hartebeest (selectivity index = −0.34), based on their availability (Figure 2). Also, Thomson's gazelle were avoided (selectivity index = −0.6), although they were the most abundant prey species (Table 2; Figure 3). These trends have been also confirmed by calculating the selection ratio ${\hat{w}}_{i}$ (see Table 3).

**TABLE 3:** Selection ratio (${\hat{w}}_{i}$) for a prey species based on the proportion of eaten prey (*o*_i_) and the abundance of prey (*π_i_*) (according to Höner et al., 2002; Manly et al., 2002; Kissui & Parker, 2004)

Species	Abundance	Proportion	Frequency of scat occurrence (*u_i_*)	Scat prop. (*o_i_*)	Selection ratio (${\hat{w}}_{i}$)	Standardized ratio (*B_i_*)	Standard error	χ^2^	*p*
Beisa oryx	1	8.01				0			
Bush buck	2	0.00	5	0.18	1155.74	0.90	15.20	1.28	.2568393
Cape hare	362	0.02	6	0.22	7.66	0.00	1.11	344.39	7.05571E−77
Dik‐dik	35	0.00							
Duiker	45	0.00							
Eland	174	0.01							
Gerenuk	16	0.00							
Giraffe	191	0.01	2	0.07	4.84	0.00	1.54	185.08	3.76444E−42
Grant's gazelle	636	0.05	7	0.25	5.08	0.00	0.83	615.30	7.8514E−136
Impala	364	0.02	2	0.07	2.54	0.00	1.11	358.04	7.50866E−80
Hartebeest	1250	0.10	2	0.07	0.73	0.00	0.57	1244.01	1.6605E−272
Lesser kudu	14	0.00	1	0.03	33.02	0.02	5.74	11.26	.000789113
Reedbuck	1	8.01							
Spring hare	693	0.05	4	0.14	2.66	0.00	0.79	681.09	3.868E−150
Steenbok	37	0.00							
Thomson's gazelle	1208	0.09	1	0.03	0.38	0.00	0.58	1205.00	4.9882E−264
Warthog	8	0.00	1	0.03	57.78	0.04	7.59	5.44	.01963066
Wildebeest	4822	0.38	1	0.03	0.09	7.54	0.24	4816.00	0
Zebra	2623	0.21	2	0.07	0.35	0.00	0.37	2614.01	0
Total	12,482	1	27			1			

Note

The standardized selection ratio (*B_i_*) is calculated as ${\hat{w}}_{i}/\left(\Sigma^{i=1}{\hat{w}}_{j}\right)$ and estimates the probability of a particular prey species *i* being selected if all prey types were equally available; standard errors and χ^2^ statistics are given.

## 4 DISCUSSION

Our results show that Grant's gazelle was the most preferred cheetah prey (Table 2). Grant's gazelle falls within the preferred cheetah weight range (23–56 kg). Preference of Grant's gazelle in the cheetah diet is not surprising given that they were also recorded as an important primary prey species in the neighboring Nairobi National Park (Eaton, 1974). The second most abundant prey item in the scats was Cape hare (Table 2). Small mammals like hares can make up a larger portion of the cheetah diet than expected as they tend to be eaten rapidly by the cheetah and are likely to escape observation than larger kills (Graham, 1966; Jeo et al., 2018). This is more likely in marginal, arid habitats, and anthropogenic landscapes, where medium‐sized ungulate prey is absent or occurs in comparatively low densities (Jeo et al., 2018). Past research on the Namibian farmlands recorded spring hare (Pedetes capensis) and hares (Leporidae) as the most important cheetah prey item outside of birth peaks of ungulates (Marker et al., 2003, 2018; Wachter et al., 2006). In Eastern Africa, cheetahs prey on hares in Maasai Mara National Reserve in Kenya and Serengeti National Park in Tanzania, although in small proportions (Broekhuis et al., 2017; Cooper et al., 2007). Although hares were consumed, such small prey is insufficient to feed family groups with growing litter cubs (Farhadinia et al., 2012). Other small mammalian prey species recorded in the cheetah diet included duiker, steenbok, spring hare, and giant rat (Table 1).

Cheetahs in the study area also showed preference for bushbuck, warthog, and kudu (Figure 3) although they were among the least abundant species recorded during the game counts (Table 1). Bushbuck has been previously recorded as a less common cheetah prey in Southern Africa while warthog is known to be a non‐regular cheetah prey in Eastern and Southern Africa, probably due to their lower species abundance (Broekhuis et al., 2017; Craig et al., 2017). In Iran, cheetahs were also observed to avoid wild boars even though they were the most abundant prey species (Farhadinia et al., 2012). Preference of kudu over other antelope species is not surprising as kudu has been previously observed as preferred cheetah prey in South Africa and Botswana where they were the most abundant antelope species available in the study area (Bissett & Bernard, 2006; Craig et al., 2017). Preference of less common prey species by cheetahs in the study area suggests that cheetahs do not only prey upon the more abundant species in order to lessen the cost of hunting but they can also hunt opportunistically for less abundant prey species.

Larger prey such as cokes hartebeest, blue wildebeest, lesser kudu, plains zebra, and giraffe Giraffa camelopardalis were also recorded in the cheetah diet (Table 2). Of the larger prey, cheetah especially showed preference for giraffe. The two cheetah scat samples containing giraffe hair were collected in Kima Ranch in 2012 and in Kapiti Plains Estate in 2013, respectively. Giraffe is not a common cheetah prey species and it is generally avoided due to its size (Hayward et al., 2006). However, cheetah has been recorded feeding on giraffes, mainly juveniles in Kwazulu‐Natal, South Africa (Hunter, 1998). Wildebeest, common zebra, and hartebeest were the most available prey species in the study area but they were the least preferred prey of cheetah (Table 2; Figure 3). Previous studies have shown that cheetahs can hunt and kill large prey while hunting in a coalition or may hunt and kill juveniles of large herbivores (Broekhuis et al., 2017; Hayward et al., 2006). Zebra has also been recorded as suitable prey utilized by cheetah in low quantities in the Kruger National Park and southern Kalahari in South Africa (Mills et al., 2004; Pienaar, 1969) and Kafue National Park in Zambia (Mitchell et al., 1965). Hartebeest, both adult and juvenile frequently occurred in the cheetah diet in Nairobi National Park (Eaton, 1974).

In this study, we found primates, such as vervet monkey Chlorocebus pygerythrus and yellow baboon Papio anubis, as well as the rock hyrax in the cheetah scat (Table 2). This is the first time that these prey species have been documented in the cheetah diet across their distribution ranges in Africa. Primates are commonly known to be preyed upon by leopards Panthera pardus but rarely by cheetah (Zuberbuehler & Jenny, 2002). Leopards have also been observed to prey on vervet monkeys and rock hyraxes in South Africa (Schwarz & Fischer, 2006). However, cheetah preying on primates and hyraxes has been documented already before. A collared female cheetah was observed to hunt and feed on a vervet monkey and tree hyrax Dendrohyrax arboreus in 2004 and 2005, respectively (Wykstra, 2015). According to Wykstra (2015), selection of primates and hyraxes by the female cheetah was due to reduced wild ungulate prey in the study area as a result of poaching. Additionally, there were few suitable habitats in the area and the cheetah was restricted to thick bushes near a settlement in the study area. This evidence suggests that smaller mammalian species which are not usually considered as cheetah prey in other areas including protected areas can contribute to the cheetah diet in anthropogenic landscapes. Therefore, anthropogenic landscapes can still be valuable cheetah habitats if sufficient smaller prey is available (Jeo et al., 2018). There was no evidence of predation on birds, especially ground‐dwelling birds in this present study. This may suggest that either cheetah did not prey on birds in the study area or the sample size was too small to approve the consumption of birds. In Northern Kenya, however, cheetahs have been reported to feed on ground‐dwelling birds, especially the vulturine guinea fowl (Acryllium vulturinum; Hamilton, 1986).

Traces of domestic stock was also present in the cheetah diet and they contributed a relatively large fraction of consumed prey (Table 2). None of the scats samples collected during this study were in response to human–wildlife conflict incidences in the study area. The samples were opportunistically collected in the different ranches within the study area during the study period. Sub‐division of former ranches in the study area and poaching has led to a decline in wild prey densities (Reid et al., 2008; Wambua, 2008). Livestock numbers in the study area are also higher due to increased human settlements in the sub‐divided ranches (Behnke, 2008). In the absence of wild prey, cheetahs in the study area may have instead preyed on livestock especially domestic goat, which was the third most preferred cheetah prey after Grant's gazelle and Cape hare (Table 2). This finding agrees to that of Farhadinia et al. (2012) where cheetahs in Iran showed preference for livestock in areas where availability of wild prey was negatively impacted by livestock grazing.

Findings of this study give basic insight of dietary preference of free‐ranging cheetahs in south‐eastern Kenya. Free‐ranging cheetahs in the study area primarily relied on wild mammalian prey as their main food source. This reflects their diversity in prey selection in rangelands outside protected areas probably due to low densities of ungulate prey. Presence of domestic stock in the cheetah scat showed that cheetahs occasionally prey on livestock thereby generating conflict with the local community.

### 4.1 Limitations of study

Our study used strip‐transect method (i.e., counting all animals within 200 m from the transect) to determine the composition and abundance of cheetah prey in the study area instead of line transect method which calculates the actual distance from the road each animal is sighted. Strip transects are likely to introduce error and bias in areas with varying visibility as it assumes that all animals are equally detectable over time (Ogutu et al., 2006). This is unlikely in the wet season when the vegetation in the study area is thicker as sighting of small animals would be more difficult due to reduced visibility.

We also analyzed a small sample size (*N* = 27) which can only give basic insight to the dietary habits of free‐ranging cheetahs in Kenya's rangelands outside protected areas. Low cheetah densities in the study area limited collection of enough cheetah scat samples during our study. Additionally, we had to remove all scat samples, which had no traces of predator hair from our analysis. This reduced our sample size because we were not able to assign these scats to cheetah with certainty without molecular analysis. We also did not apply any correction factors due to the small sample of identified cheetah scat. Therefore, caution should be taken with interpretation of these results as the number of scats produced by cheetah for different prey animals vary depending on the prey's body size and its ratio of fur and meat (Wachter et al., 2006). For instance, small prey gives a higher number of field‐collectable scats because they are composed of relatively more indigestible matter (fur). This leads to over‐estimation of the small prey species consumed by the cheetah (Marker et al., 2003). We also excluded prey species such as vervet monkeys, baboons, rock hyraxes, giant rat, and domestic stock (goat, sheep, and cow) from the preference and Jacob Index analysis because their abundance was not recorded during the game counts although they were present in the diet. Therefore, the results of this study should be interpreted with caution due to these limitations of our data. Nevertheless, our results might provide valuable and first data on diet preferences of a free‐ranging cheetah population living in rangelands outside protected areas in Kenya.

## AUTHOR CONTRIBUTION

**Noreen Mutoro:** Conceptualization (equal); Data curation (equal); Formal analysis (equal); Methodology (equal); Visualization (equal); Writing—original draft (equal); Writing—review & editing (equal). **Robert Chira:** Conceptualization (equal); Methodology (equal). **Nathan Gichuki:** Data curation (equal); Methodology (equal); Project administration (equal); Supervision (equal). **Edward Kariuki:** Methodology (equal). **Jonas Eberle:** Formal analysis (equal); Methodology (equal). **Jan Christian Habel:** Methodology (equal); Writing—original draft (equal); Writing—review & editing (equal). **Mary Wykstra:** Conceptualization (equal); Data curation (equal); Funding acquisition (equal); Methodology (equal); Project administration (equal); Writing—original draft (equal); Writing—review & editing (equal).

## Supporting information

Table S1

## Data Availability

All data of this manuscript will be available as Table S1 in online appendices.
